# On variation and uncertainty

**DOI:** 10.1007/s40037-016-0281-5

**Published:** 2016-07-12

**Authors:** Jimmie Leppink, Patricia O’Sullivan, Kal Winston

**Affiliations:** 1 University Maastricht, Maastricht, the Netherlands; 2 University of California, San Francisco, USA; 3 The Commonwealth Medical College, Pennsylvania, USA

The overall purpose of the ‘Statistical Points and Pitfalls’ series is to help readers and researchers alike increase awareness of how to use statistics and why/how we fall into inappropriate choices or interpretations. We hope to help readers understand common misconceptions and give clear guidance on how to avoid common pitfalls by offering simple tips to improve your reporting of quantitative research findings. Each entry discusses a commonly encountered inappropriate practice and alternatives from a pragmatic perspective with minimal mathematics involved. We encourage readers to share comments on or suggestions for this section on Twitter, using the hashtag: #mededstats.

Studies regularly report the *p*-value from a statistical significance test with incomplete or no information that conveys meaning regarding the magnitude of the difference of interest revealed by the data. This is unfortunate, because – contrary to the *p*-value – that omitted information can help researchers understand the results and, if needed, perform analyses of statistical power to determine required sample size for subsequent studies [1]. Moreover, a *p*-value says nothing about the probability that a null hypothesis or its alternative is true or that findings are due to chance, and cannot serve as an indicator of the importance of a result [2]. Therefore, in this entry, we recommend reporting means and standard deviations (or other appropriate measures of centrality and variation), and confidence intervals when interested in generalizing from sample to population. This essay also clarifies the need for due caution in interpreting *p*-values and confidence intervals.

## A fair lottery

A frequent misconception is that the *p*-value is the probability that findings are due to chance (alone) or – put in other words but generally meaning the same – the chance that the null hypothesis (*H*_0_) is true [2]. Let us illustrate this misconception with a simple example. Suppose, you bought one ticket for a lottery for which one million tickets have been sold. Assuming a fair lottery (*H*_0_), every ticket has the same chance of winning and thus your chance of winning, *p*, is 1 in a million (provided, of course, that the winning ticket is among those sold). This *p*-value, for any single ticket, is a conditional probability – the probability of any single ticket winning the lottery given the assumption (the condition) that *H*_0_ (fair lottery) is true: *p*(winning | *H*_0_) = 0.000001. Of course, one of the tickets must win the lottery. So, after the lottery is drawn, you now have the winning ticket, which *a priori* had a chance of winning of 1 in a million. However, this does not imply that you can now determine that the probability that the lottery was fair given that you have the winning ticket, *p*(*H*_0_ | winning), is 0.000001; the mere fact that your ticket was drawn contains no information about the fairness of the lottery (how it was conducted, who made the draw, etc.). Indeed, if we have assumed the null hypothesis that the lottery was completely fair, then the probability that your ticket was drawn by chance must be 1 (100 %). Null hypothesis significance testing always assumes random sampling, and under that assumption, in our example, the probability that findings are due to chance is always 1 (100 %). Take for instance a baseline difference between randomized groups in an experiment: this difference is entirely the result of chance, regardless of the *p*-value of a statistical test on this difference.

## If the null hypothesis is true

In the context of empirical research, the *p*-value is the probability of getting the data we found (or more specifically: the value of our test statistic) or further away from *H*_0_ given the assumption that *H*_0_ is true, analogous to *p*(winning | *H*_0_) in the example above. Associating low *p*-values (e. g. *p* < 0.001) with ‘this cannot be chance’ can come together with the misconception that a mean difference observed in a sample must be about the same as in the population. This can be erroneous as *p*-values can vary substantially from sample to sample [3], especially in smaller samples [1]. For example, if we test Pearson’s linear correlation coefficient (*r*) between performance and intrinsic motivation, *H*_0_ is *r* = 0 (i. e., there is no linear relation). The *p*-value for this coefficient only indicates what proportion of all samples of the same size drawn from the same population would result in the same correlation coefficient or further away from zero *if the null hypothesis is true*. In other words, the *p*-value is *p*(observed correlation coefficient or more extreme | *H*_0_). Therefore, we cannot say that the null hypothesis is true based on the *p*-value or how likely *H*_0_ is given the data, *p* (*H*_0_ | observed correlation coefficient or more extreme), or about the uncertainty around the observed correlation coefficient.

## Confidence intervals

A concept that plays an important role in *p*-values as well as in confidence intervals is that of the *standard error*. The standard error estimates the extent to which an estimator – such as a mean difference, a standard deviation or a correlation coefficient – varies across samples of the same size drawn from the same population. Many articles report 95 % confidence intervals. For instance, a researcher could construct a 95 % confidence interval around the observed difference in average performance scores when comparing Approach A and Approach B. This interval takes the observed difference in average performance scores and extends about twice the standard error to either side of the mean difference (i. e. in the case of a normal distribution, the interval of middle point plus/minus two standard errors includes about 95 % of all possible values [4]). The 95 % confidence interval corresponds with a two-sided statistical test (null hypothesis, *H*_0_: means are equal; alternative hypothesis, *H*_1_: means are different) with the conventional *α* = 0.05 as significance level. That is, if the 95 % confidence interval of the difference between two means does *not *include the value specified under *H*_0_ (‘0’ in our case of ‘means are equal’ for Approach A and Approach B) your statistical software programme will also give a *p*-value below 0.05; if the interval includes the value specified under *H*_0_, however, the *p*-value will be larger than 0.05. Thus, like the *p*-value, the 95 % confidence interval indicates whether an observed difference – in this case: a difference in means – is statistically significant at the *α* = 0.05 significance level.

## What confidence intervals tell us that *p*-values cannot

The 95 % confidence interval provides an indication which the *p*-value cannot tell us: the uncertainty around observed differences or the extent to which the difference of interest may vary from sample to sample [3]. For instance, if the mean difference between Approach A and Approach B is 3 and the standard error is 1, the 95 % confidence interval extends from about 1 to 5. If the standard error is 0.5 instead of 1, the 95 % confidence interval extends from about 2 to 4. Both intervals indicate that the observed difference of 3 is statistically significantly different from 0 at the *α* = 0.05 level since 0 is not in the interval. However, we can expect more sample to sample fluctuation in the case of an interval from 1 to 5 than in the case of an interval from 2 to 4. Remember also that means and other estimators fluctuate more with small samples.

## Replication is fundamental to science

Unfortunately, misconceptions about *p*, such as claims that it is the chance that *H*_0_ is true or the probability that findings are due to chance [2], lead a good number of researchers to misinterpret *p*-values and to erroneously believe that *p*-values inform us about the likelihood of observing findings we obtained in a current sample in a new sample [3]. However, Cumming [3] states (*p*. 286): ‘Replication is fundamental to science, so statistical analysis should give information about replication. Because *p*-values dominate statistical analysis […], it is important to ask what *p* says about replication. The answer to this question is ‘surprisingly little’.’ In a simulation of 25 replications of a typical experiment drawn from the same population, the *p*-value varied from below 0.001 to 0.76, illustrating that the *p*-value is a notoriously unreliable measure. For more details, we refer to Cumming’s paper [3], which is a great read for researchers who associate *p* with certainty or with confidence around findings.

Researchers consequently should not use qualifiers such as we commonly see: 0.05 as indicating that the null hypothesis is ‘quite unlikely’, *p*-values of 0.01 as indicating that the null hypothesis is ‘really unlikely’, and *p*-values of 0.001 and below as indicating that the null hypothesis is ‘really, really unlikely’ (because the *p*-value is ‘very significant’). Such qualifiers stimulate erroneous beliefs that we can be ‘quite sure’ (*p* < 0.05), ‘really sure’ (*p* < 0.01) or even ‘almost absolutely sure’ (*p* < 0.001) that *H*_0_ is not true. These statements also run counter to the recommendation in a recent statement on *p*-values by the American Statistical Association (ASA) to not base scientific conclusions only on whether a *p*-value passes a specific threshold [2]. In fact, given that confidence intervals such as *p*-values provide information on statistical significance and provide additional information, we might as well stop using *p*-values altogether.

## Confidence intervals express uncertainty not confidence

In line with misconceptions about *p*, a 95 % confidence interval is sometimes interpreted as the chance that *H*_0_ is true (when the interval includes the value under *H*_0_) or the chance that the interval includes the actual population value (e. g. mean difference) of interest. The term ‘confidence’ is misleading; it would be more appropriate to speak of an estimation interval (emphasizing uncertainty around point estimates) or testing interval (given the link with *p*). Provided that assumptions underlying our statistics are realistic [4], a 95 % interval includes the true population value of interest in 95 % of all possible samples of the same size drawn from the same population. Statistically significant or not, a very wide confidence interval indicates that when drawing new samples of the same size from the same population, we may well obtain results that are very different from those we observe in the sample at hand. Contrary to *p*-values, confidence intervals give useful information about replication: Cumming and colleagues [5] demonstrated that a replication sample gives a mean that falls within the 95 % confidence interval from the initial study in about 83 % of the cases. In other words, in 17 % of the cases, the mean observed in a replication sample will *not* fall in the 95 % confidence interval from the initial study. Hence, a 95 % confidence interval can be regarded as an 83 % prediction interval for a replication mean. This kind of information cannot be derived from a *p*-value by any means.

## To conclude

Statistics is about dealing with uncertainty. While *p*-values indicate whether or not a difference or relation of interest is statistically significant, they tell us nothing about the likelihood of the null hypothesis being true or that the findings are due to chance, and also cannot be used as an indicator of the importance of findings. Confidence intervals, such as *p*-values, indicate whether or not a finding is statistically significant. However, confidence intervals also remind us of the uncertainty around sample estimates and provide some indication of what might happen when replicating a study at hand. So what do we recommend?

1. Always report descriptive statistics of centrality and variation and never a *p*-value alone.
2. When interested in generalizing findings from sample to population, preferably report confidence intervals rather than *p*-values.
3. Carefully consider the width of the confidence interval when interpreting the meaning of the results.
4. Avoid statements asserting null hypotheses to be true (or not), or forming conclusions about significant findings based on the magnitude of the *p*-value.
